# Identification of a protein responsible for the synthesis of archaeal membrane-spanning GDGT lipids

**DOI:** 10.1038/s41467-022-29264-x

**Published:** 2022-03-22

**Authors:** Zhirui Zeng, Huahui Chen, Huan Yang, Yufei Chen, Wei Yang, Xi Feng, Hongye Pei, Paula V. Welander

**Affiliations:** 1 Department of Ocean Science and Engineering, Southern University of Science and Technology, Shenzhen, 518055 China; 2 Southern Marine Science and Engineering Guangdong Laboratory (Guangzhou), Guangzhou, 511458 China; 3 State Key Laboratory of Biogeology and Environmental Geology, Hubei Key Laboratory of Critical Zone Evolution, School of Geography and Information Engineering, China University of Geosciences, Wuhan, 430074 China; 4 Department of Earth System Science, Stanford University, Stanford, CA 94305 USA

**Keywords:** Archaeal genetics, Archaeal physiology, Membrane lipids, Metabolic pathways

## Abstract

Glycerol dibiphytanyl glycerol tetraethers (GDGTs) are archaeal monolayer membrane lipids that can provide a competitive advantage in extreme environments. Here, we identify a radical SAM protein, tetraether synthase (Tes), that participates in the synthesis of GDGTs. Attempts to generate a tes-deleted mutant in Sulfolobus acidocaldarius were unsuccessful, suggesting that the gene is essential in this organism. Heterologous expression of tes homologues leads to production of GDGT and structurally related lipids in the methanogen Methanococcus maripaludis (which otherwise does not synthesize GDGTs and lacks a tes homolog, but produces a putative GDGT precursor, archaeol). Tes homologues are encoded in the genomes of many archaea, as well as in some bacteria, in which they might be involved in the synthesis of bacterial branched glycerol dialkyl glycerol tetraethers.

## Abstract (web summary)

The cell membranes of many archaea contain characteristic membrane-spanning lipids known as glycerol dibiphytanyl glycerol tetraethers. Here, Zeng et al. identify a protein that participates in a key step of the synthesis of these lipids from diether precursors.

## Introduction

One of diagnostic features that distinguishes archaea from bacteria and eukaryotes is the chemical composition of their membrane lipids. Archaeal membranes are mainly composed of two types of isoprenoid ether lipids: glycerol diphytanyl diethers (archaeol) and glycerol dibiphytanyl glycerol tetraethers (GDGTs)^1^. Archaeol forms a bilayer membrane that is similar to bacterial and eukaryotic fatty acid bilayers, while GDGTs are membrane-spanning lipids that result in a monolayer membrane^2^. Various archaeal groups contain different proportions of archaeol and GDGTs in their membranes. For instance, haloarchaea and some methanogens are found to produce only archaeol, while thermophilic crenarchaea have GDGTs as their dominant membrane lipids^3^. From a physicochemical aspect, the formation of monolayers results in greater membrane rigidity and lower permeability which might provide a structural advantage to archaeal cells in extreme environments^4^. Culture experiments indicate that archaea elevate the proportion of tetraether over diether lipids (GDGT/archaeol ratio) under high growth temperature to warrant membrane thermal stability^5,6^. This observation is also in agreement with liposome studies showing that GDGT-based liposomal membranes are more stable than archaeol-based membranes^7^. In addition, liposomal transport studies show that GDGTs significantly reduce the membrane permeability of water, proton, ammonia, urea, and glycerol compared to archaeol^7,8^. This low membrane permeability is proposed to provide an energetic advantage for archaea in chronic energy stress environments^2^.

Some archaea can further modify the GDGT structure by introducing cyclopentane and cyclohexane rings, hydroxyl or methyl moieties, altering the polar head groups, or cross-linking the two biphytanyl chains^3^. Such structure variability contributes to the overall complexity of the archaea lipidome, allowing GDGT profiles to be taxonomic markers for archaeal community analyses^9^. Moreover, GDGT modification generally provides additional rigidity or flexibility to adapt to environmental fluctuations, and these lipid modifications, in turn, can reflect environmental conditions^10–13^. Because fossilized GDGTs can be well preserved in sediments over a 100-million years old, these GDGT patterns (GDGT structures and their contents) can serve as paleoproxies that record environmental change over large time scales^14,15^.

Identification of the GDGT biosynthesis pathway is fundamental to the study of the physiological function of GDGTs as well as to the interpretation of GDGT-based biomarkers. Currently, the biochemical mechanism and the protein(s) required in the key step of core GDGT formation, from diether to tetraether, remain unknown^16^. In this study, we utilized a comparative genomics approach coupled with gene heterologous expression and lipid analyses to identify a GDGT synthase protein (tetraether synthase, Tes). Our results demonstrate that heterologous expression of Tes candidates in *Methanococcus maripaludis*, which does not produce GDGTs, resulted in the formation of GDGT, glycerol trialkyl glycerol tetraether (GTGT), and macrocyclic archaeol. Further, bioinformatics analyses demonstrated that Tes homologs are widely distributed in archaeal phyla, and are also present in some bacterial groups, possibly shedding light into the biosynthesis of the mysterious bacterial branched glycerol dialkyl glycerol tetraethers (brGDGTs).

## Results

### Identification of a GDGT synthesis protein in methanogens

We hypothesized that a potential tetraether synthase (Tes) that catalyzes the condensation of archaeol to GDGT would be a radical S-adenosylmethionine (SAM) protein, because radical SAM proteins are capable of catalyzing challenging C–C bond formation^17^. Previous studies have shown that this class of proteins are required for GDGT modifications such as cyclization and calditol head group formation in *Sulfolobus acidocaldarius*^18,19^, and are likely involved in the methylation of GDGT-related lipids in methanogens^20^. In addition, C–C bond formation of the bacterial membrane-spanning lipid diabolic acid has been proposed to occur through a radical mechanism^21^. We searched the *S. acidocaldarius* genome for all annotated radical SAM proteins (Pfam: PF04055) and, among the 18 proteins identified, Saci_0703 was the most promising candidate. Firstly, homologs of this protein (*e*-value < 1e^−50^; identity >30%) are only present in the genomes of archaea that produce GDGTs and absent from those that do not. Secondly, the gene that encodes the Saci_0703 homolog in *Methanosarcina acetivorans* C2A (MA_1486) is localized next to a ferredoxin (MA_1485) required to activate geranylgeranyl reductase (GGR; MA_1484)^22^, which catalyzes the saturation of ether lipid double bonds to form archaeol^23^, a putative precursor of GDGT. Thirdly, the *saci_0703* gene is highly expressed under all growth conditions in *S. acidocaldarius* (Supplementary Table 1). As GDGTs are the major membrane lipids present in *S. acidocaldarius*^24^, high expression of any GDGT synthesis gene would be necessary to meet this need.

We first attempted to identify the function of Saci_0703 through gene deletion analysis in *S. acidocaldarius*. However, we were unable to generate a *saci_0703* gene deletion mutant suggesting that this gene may be essential for growth. We next attempted heterologous expression of *saci_0703* in the methanogen *Methanococcus maripaludis*, which does not synthesize GDGTs, does not contain a Saci_0703 homolog, and produces the putative GDGT precursor archaeol^25^. Yet, the expression of *saci_0703* in *M. maripaludis* did not produce any GDGTs, perhaps due to the codon bias of expressing a Crenarchaeota gene in a Euryarchaeota host or to a lack of expression of a protein from a thermophile in a mesophile. We decided to express Saci_0703 homologs from more closely related organisms to the *M. maripaludis* host and searched for homologs in 322 methanogen genomes in the Joint Genome Institute (JGI) Integrated Microbial Genomes (IMG) database. We found that 172 genomes contain one Saci_0703 homolog, 22 genomes contain two copies, and 128 genomes do not contain any homologs (*e*-value  < 1e^−50^; identity >30%). We chose to express one homolog from *Methanococcus aeolicus* Nankai-3 (Maeo_0574), and two homologs from *M. acetivorans* C2A (MA_1486 and MA_1114).

**Fig. 1:**
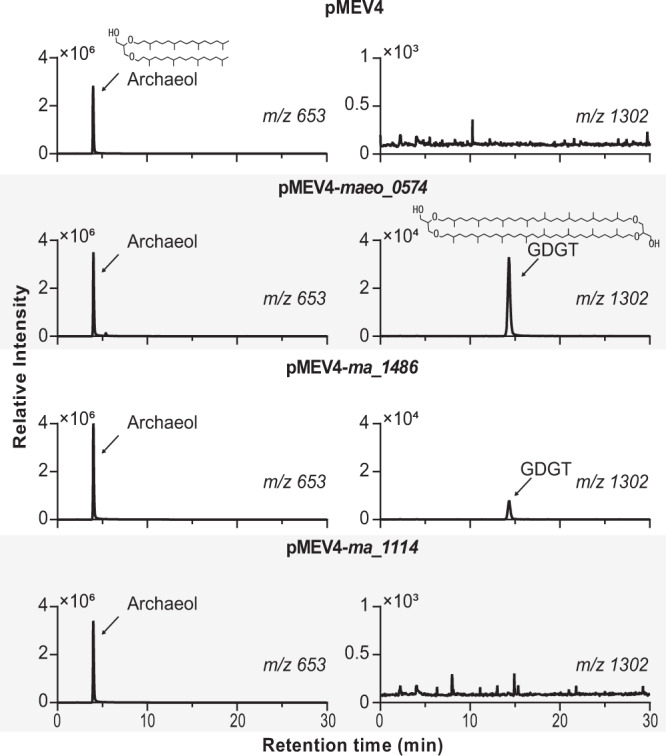
Tes (Maeo_0574 or MA_1486) is sufficient for GDGT synthesis. LC-MS extracted ion chromatograms of acid hydrolyzed lipid extracts of *M. maripaludis* wild type (WT) with empty plasmid pMEV4 or *ma_1114* heterologous expression showing only production of archaeol, and *M. maripaludis* WT with *maeo_0574 or ma_1486* heterologous expression producing GDGT. Mass spectra are shown in Supplementary Fig. 1. Source data are provided as a Source Data file.

**Fig. 2:**
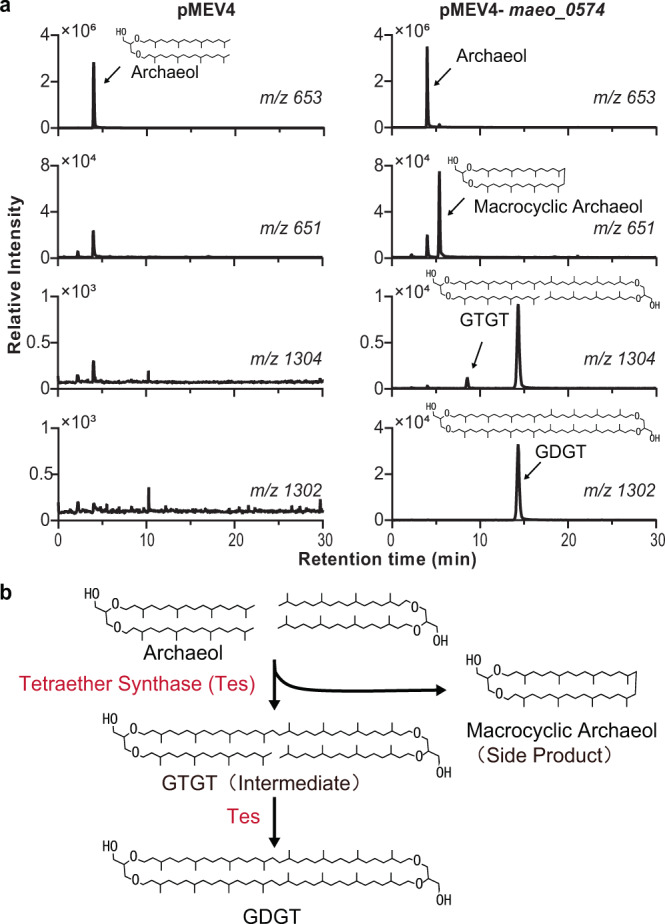
GDGT biosynthesis pathway, intermediate, and side product. **a** LC-MS extracted ion chromatograms of acid hydrolyzed extracts from heterologous expression strains showing that Tes (*maeo_0574*) can generate not only GDGT, but also the potential biosynthetic intermediate GTGT, and the side product macrocyclic archaeol. **b** Proposed GDGT biosynthesis pathway based on the Tes heterologous expression products. The chromatograms of all Tes homologs expression strains are shown in Supplementary Fig. 2, and mass spectra are shown in Supplementary Fig. 1. Notice that the double bonds on archaeol may be required for the condensation reaction (see discussion). Source data are provided as a Source Data file.

**Fig. 3:**
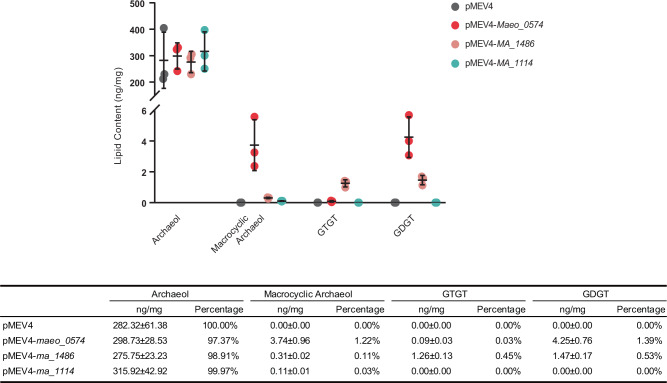
Quantification of lipids formation during Tes expression in *M. maripaludis*. The abundance of core GDGT-related lipids (ng/mg dry cell mass) from heterologous expression of Tes genes *maeo_0574*, *ma_1486*, or *ma_1114*, showing *maeo_0574* is more efficient to synthesize GDGT and related lipids than the other homologs. The abundance of core lipids was calculated relative to the peak area of the synthetic C46 GTGT internal standard (5). Results are the mean ± standard error from three biological replicates. Source data are provided as a Source Data file.

The methanogen genes encoding each hypothesized Tes homolog were expressed individually on a self-replicating plasmid in *M. maripaludis*. Expression of *maeo_0574* or *ma_1486* alone resulted in the production of GDGT (Fig. 1), indicating that the Tes protein is necessary to synthesize GDGTs. In addition to GDGT production, expression of these two Tes homologs in *M. maripaludis* also generated glycerol trialkyl glycerol tetraether (GTGT) and macrocyclic archaeol (Fig. 2a and Supplementary Fig. 2). However, expression of *ma_1114*, the second Tes homolog in the *M. acetivorans* genome, did not result in GDGT production but did produce trace amounts of macrocyclic archaeol. Further lipid quantification showed that expression of *maeo_0574* or *ma_148*6 produced tetraether lipids accounting for approximately 1.42% and 0.98% of total membrane lipids, respectively. The strain expressing *ma_1114* only produced macrocyclic archaeol at approximately 0.03% of total membrane lipids (Fig. 3). It is currently unclear if the differences observed in GDGT production by the various Tes homologs tested is due to functional or biochemical variations between homologs or results from differences in expression level of each protein in the heterologous system.

The structure of GTGT is like that of GDGT, except one of the C_40_-biphytanyl chains is replaced by two C_20_-phytanyl chains (Fig. 2). GTGT had been proposed as an intermediate in GDGT biosynthesis^1^, a consequence of incomplete side chain condensation. The co-occurrence of GTGT and GDGT when we heterologously expressed Tes suggests that this GTGT intermediate hypothesis may be correct (Fig. 2b). The production of macrocyclic archaeol was unexpected, as this lipid has only been detected in a few archaeal species^26–28^. To confirm the structure of the macrocyclic archaeol, we purified this compound from *M. maripaludis* expressing *maeo_0574*. We subsequently cleaved the ether groups through an ether cleavage reaction and analyzed the resulting products through gas chromatography-mass spectrometry (GC-MS) (Supplementary Fig. 1). The detection of biphytane with C_40_ chains rather than the C_20-_phytane, indicates macrocyclic archaeol was indeed synthesized by Tes. We propose that macrocyclic archaeol is a side product of the heterologous expression of Tes homologs in *M. maripaludis* (Fig. 2a). Together, the biosynthesis of GDGT, GTGT, and macrocyclic archaeol suggest that Tes can introduce head-to-head linkages between two phytanyl chains of archaeol.

We next wanted to determine if Tes had any potential transmembrane helices as it could provide some insight into whether this was a membrane-bound protein and where the Tes condensation reaction may be occurring in the cell. We first used Phyre2^29^ to predict whether four Tes homologs, Saci_0703, Maeo_0574, MA_1486, and MA_1114, had any transmembrane helices. Phyre2 predicted that the Saci_0703 and MA_1486 homologs contain one transmembrane helix but Maeo_0574 and MA_1114 do not contain any. We also searched for transmembrane helices through TMHMM 2.0^30^, which predicted that none of these proteins had transmembrane helices. Thus, it remains unclear whether these proteins are integral membrane proteins, membrane-associated proteins, or cytoplasmic proteins. Given their role in modifying key membrane lipids in archaea, we hypothesize that Tes homologs are at least membrane associated but this, of course, requires biochemical and structural characterization.

### Distribution of Tes in microbial genomes

**Fig. 4:**
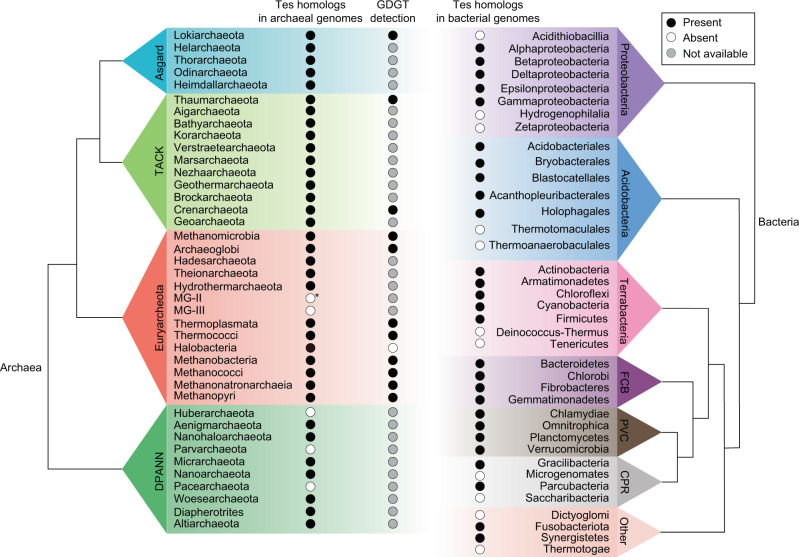
Tes homologs are found in diverse archaeal and bacterial phyla. These data were generated through BLASTP searches of Tes homologs (*e*-value < 1e^−50^, identity > 20%) in the NCBI database and searching published studies demonstrating GDGT production from pure cultures^3,5,9,10,36,69–71^. At least one strain or one genome in the phylum, class or order containing the Tes homolog and GDGTs (at least GDGT-0) is marked with a black circle. Otherwise, archaea or bacteria without any Tes homolog or GDGTs are marked with white circles. Uncultured archaeal strains or with cultured strains not yet tested for GDGT production, are marked with gray circles. Only one putative Tes homolog (*e*-value = 6e^−53^, identity = 28%) was identified from 231 MG-II MAGs (*). Phylogenetic lineages of archaea and bacteria are modified from references^31,72,73^. Proteobacteria and acidobacteria are divided into class level and order level, respectively. Terrabacteria, FCB (Fibrobacteres-Chlorobi-Bacteroidetes), PVC (Planctomycetes-Verrucomicrobia-Chlamydiae) and CPR (Candidate Phyla Radiation) are bacterial superphyla^72,73^ and only several phyla are shown in each group. Bacterial phyla that do not belong to these groups are assigned “Other” in the figure. Details and references are listed in Supplementary Table 2.

Understanding the predominant biological source of GDGTs in modern environments is one of the fundamental constraints necessary for the proper interpretation of GDGT-based proxies, such as the TEX_86_ (tetraether index of 86 carbons) sea-surface temperature proxy^15^. Our identification of the Tes protein allows us to better understand the distribution of GDGT producers through the analyses of genomic and metagenomic databases. To do so, we performed a Basic Local Alignment Search Tool (BLASTP) search (*e*-value < 1e^−50^; percent identity >30%) of protein sequences to identify Tes homologs in the National Center for Biotechnology Information (NCBI) database. Using the *M. acetivorans* MA_1486 homolog as a search query, we found that Tes homologs are widely distributed in all three archaeal superphyla (Asgard, TACK, and DPANN) and the Euryarchaeota. Only 3 (Huberarchaeota, Parvarchaeota, and Pacearchaeota) out of 27 archaeal phyla^31^ do not contain a Tes homolog (Fig. 4 and Supplementary Figs. 3–5 and Supplementary Table 2) suggesting these archaea do not produce GDGTs.

More relevant to the TEX_86_ paleotemperature proxy, we searched for Tes homologs in the metagenome-assembled genomes (MAGs) of Marine Group II (MG-II) and Marine Group III (MG-III) Euryarchaeota. The major producers of GDGTs in marine water columns have been suggested to be the Marine Group I (MG-I) Thaumarchaeota – providing a critical source constraint for TEX_86_ paleoproxies^19^. However, 16S rRNA analyses of various open ocean settings have identified the MG-II Euryarchaeota as another potential source of GDGTs in the upper water column which could skew the TEX_86_ signal^32^. Our bioinformatic search for Tes homologs in MG-II archaea revealed that only 1 MG-II MAG (NCBI Genome ID: GCA_002457195.1) out of 231 MG-II MAGs was found to have a Tes homolog. Further, we did not identify any Tes homologs in Marine Group III (MG-III) MAGs, another group of uncultured marine Euryarchaeota generally considered low-abundance members of deep mesopelagic and bathypelagic communities^33^. These data suggest that both MG-II and MG-III do not produce any GDGTs and are most likely not a significant contributor to open ocean GDGT pools.

Interestingly, 20 Halobacteria genomes, for instance, *Halobellus clavatus* and *Halorientalis regularis*, were found to have Tes homologs. However, previously published lipid analyses of these strains did not find any GDGT production^34^ so the function of these Halobacterial Tes homologs remains unclear. We also observed Tes homologs in the MAGs from the DPANN archaea which were unexpected as most archaea belonging to this group are thought to be symbionts whose genomes lack lipid biosynthesis genes^35^. The one cultivated DPANN species, *Nanoarchaeum equitans*, has been found to contain GDGTs but these were thought to be acquired from its Crenarchaeota host, *Ignicoccus hospitalis*^36^. In support of this, we find that the *N. equitans* genome does not harbor a Tes homolog while the *I. hospitalis* genome does. Given this pattern, it is possible that Tes homologs identified in DPANN MAGs may belong to their symbiotic host.

Finally, we also identified 456 Tes homologs in the Bacterial domain such as the Acidobacteria and various Proteobacteria (Fig. 4 and Supplementary Fig. 4). To date, no bacteria have been shown to produce GDGTs so it would seem unlikely that these bacterial Tes homologs are condensing diethers to GDGT. Rather we hypothesize that these bacterial homologs may be responsible for the synthesis of membrane-spanning lipids in bacteria such as the ether-linked brGDGTs^37^. Geochemists first identified brGDGTs in soils and peats^38^ but they have also been found in a variety of terrestrial and marine environments^39,40^. Bacterial brGDGTs are unique lipids in that they harbor a mixture of archaeal and bacterial properties. Although the ether linkages to glycerol along with the monolayer structure and cyclopentane moieties are reminiscent of archaeal membranes, the alkyl moieties of brGDGTs are not isoprenoidal but rather composed of branched carbon chains suggesting a bacterial source^38,41^. Acidobacteria are thought to be one source of brGDGTs and have been shown to produce small amounts of brGDGTs with no rings^42^. A recent study confirmed that the Acidobacterial strain, *Edaphobacter aggregans*, produces brGDGTs when grown under conditions of oxygen limitation^43^. We found that *E. aggregans* contains one Tes homolog (identity = 32%, *e*-value = 4e^−61^) in its genome, and that the top two bacterial Tes homologs with a sequence identity of 42% (*e*-value = 1e^−149^) and 40% (*e*-value = 1e^−139^) are from Acidobacterial species *Candidatus Koribacter versatilis* Ellin345 and *Candidatus Solibacter usitatus* Ellin6076, respectively, suggesting that these proteins may be involved in brGDGTs biosynthesis.

## Discussion

Archaeal GDGT membrane lipids have been studied for decades^2,3^, yet the full biosynthetic pathway for synthesizing these membrane-spanning tetraethers has remained unclear. Here, we identify a radical SAM protein, Tes, required for the conversion of two diethers to one tetraether, unraveling a long-standing puzzle of GDGT biosynthesis. The discovery of Tes is significant for understanding the biochemical mechanisms behind GDGT formation as well as having implications for our interpretation of GDGT-based paleotemperature proxies.

The mechanistic details of how the unusual C-C bond formation occurs between two diethers to generate a tetraether have been difficult to ascertain. One area of uncertainty has been whether the diether precursor for GDGT biosynthesis is fully saturated or unsaturated^16^. Feeding studies with deuterium-labeled lipid precursors have suggested that the presence of a ∆^14–15^ double bond in the proposed digeranylgeranylglycerol phosphate (DGGGP) precursor was crucial for the formation of GDGTs^44^ while other studies have suggested that the diether precursor is fully saturated^45^. While the results we present here cannot distinguish whether the diether precursor is fully saturated or not, we can glean some insight into the potential mechanism of GDGT formation through the protein domain features of Tes. This protein contains two CxxxCxxC motifs^17^, the characteristic signature of all radical SAM proteins, suggesting Tes could bind two iron-sulfur clusters and may harbor two distinct active sites with separate catalytic functions. One site could potentially catalyze the desaturation of a fully saturated diether precursor^46^ or mediate double bond migration to generate a terminal olefin^47^. The second site could then generate a C-C bond between sp^2^ and sp^3^ carbon center^48,49^. The coordination of two active sites could trigger the head-to-head condensation of two diether lipids to form one GDGT. The discovery of Tes now allows for biochemical and structural characterization that could elucidate this potentially novel radical mechanism for C-C formation.

In addition to the question of whether the Tes substrate is saturated or unsaturated, it is also unclear if the configuration of the glycerol head groups is relevant to the Tes mechanism. There are two glycerol configurations of GDGTs in nature, the parallel and antiparallel GDGTs^50,51^. Gräther and Arigoni^50^ showed that some archaeal strains, such as *Methanothermobacter marburgensis* (DSM 2133), *Thermoplasma acidophilum*, and *Saccharolobus solfataricus* P1, produce approximate 1:1 ratio of these two configurations. We found that each of these genomes contains only one Tes homolog. Therefore, it is very likely that one Tes protein is able to generate both parallel and antiparallel GDGTs, and at least some Tes homologs have no preference for the orientation of the glycerol headgroups during head-to-head condensation.

Further studies of Tes could also provide insight into the interplay between the various GDGT biosynthesis proteins discovered thus far. Tes is the third radical SAM protein, in addition to calditol synthase (Cds)^18^ and the GDGT ring synthase (Grs)^19^, found to be involved in GDGT biosynthesis. Archaea seem to employ radical mechanisms readily in the modification of their membrane lipids and we hypothesize that other GDGT derivatives, such as the H-shaped GDGTs or GDGTs with an unusual cyclohexane ring^3^, could also be generated through radical mechanisms catalyzed by radical SAM proteins. In addition, it has been proposed that GDGT formation and modification may have a sequential component that allows the cell to balance its membrane structural needs with the need for redox balance under chronic energy stress conditions often brought on by the extreme environments many archaea inhabit^2,48^. Thus, a biosynthetic scheme where the cell balances condensation by Tes, Grs cyclization, and saturation by the geranylgeranyl reductase (GGR) could serve a role in the cell’s effort to maintain physiological homeostasis^48^. Understanding how these proteins are coordinated and how the entire mechanism of GDGT formation and modification is regulated could provide significant insight into the physiological and environmental factors that influence GDGT formation. The discovery of Tes now allows for direct testing of these hypotheses.

The discovery of Tes also aids in our efforts to better constrain GDGT-based paleotemperature proxies. The TEX_86_ proxy was the first proxy to be based on archaeal lipids and was established as a ratio of the relative abundance of specific cyclized GDGTs in surface sediments that correlated with annual mean sea surface temperature (SST)^15^. While the connection between GDGT cyclization and temperature continues to be debated, elucidating the GDGT biosynthesis pathway has allowed for a better grasp of the sources of GDGTs in open oceans. We have previously demonstrated that the lack of GDGT ring synthase (Grs) homologs in MG-II genomic datasets suggests that these archaea, which have not been cultured, did not produce any cyclized GDGTs^19^. Here, we demonstrate that the MG-II Euryarchaeota also do not contain a Tes homolog suggesting that they do not produce any GDGTs at all. These bioinformatic analyses further confirm that the dominant source of GDGTs in the open ocean are the MG-I Thaumarchaeota, although culturing and lipid analyses of MG-II Euryarchaeota are necessary to unequivocally confirm that these archaea do not produce any GDGTs.

Finally, the identification of Tes homologs in many bacterial genomes provides potential protein candidates for the formation of membrane-spanning brGDGT lipids in bacteria (Fig. 4 and Supplementary Fig. 4). As mentioned above, brGDGTs were first discovered in a variety of environments and were proposed to be of bacterial origin due to the chemical nature of their alkyl chains and to their linkage to glycerol-3-phosphate rather than glycerol-1-phosphate, as is observed in the archaeal GDGTs^37^. Additionally, geochemical studies have found a statistical relationship between brGDGTs distribution in sediments and changes in pH and temperature which has resulted in the development of brGDGT-based paleoproxies that help reconstruct past environmental pH and temperatures^52^. However, it has been difficult to properly constrain brGDGT paleoproxies because bacteria that produce significant amounts of these lipids, and the proteins required for their biosynthesis, have not been well identified^42^. It has been proposed that the synthesis of brGDGTs occurs through the head-to-head condensation of two iso-C15:0 branched phospholipids^37^. Branched chained fatty acids (BCFAs) such as iso-C15:0 are produced by a variety of bacteria and are characterized by a single methyl group at the penultimate (iso) or antepenultimate (anteiso) carbon^53^. Given that the tails of the bacterial iso-branched fatty acids and the archaeal isoprenoid lipids are structurally identical, it is possible that these bacterial Tes homologs are involved in the formation of brGDGTs. Follow-up studies demonstrating the biochemical activity of these bacterial Tes homologs can be carried out to verify if they are true brGDGTs biosynthetic enzymes and, if confirmed, can provide insight into the potential sources of these lipids in a variety of ecosystems and allow for a more robust interpretation of brGDGT paleoproxies.

## Methods

### Microbial strains, media, and growth conditions

Strains used in this study are listed in Supplementary Table 1. *M. maripaludis* S001^54^ and its derivatives were grown in McF medium under a gas phase of N_2_/CO_2_ (80:20) at 37 °C^55^, and the solid medium was added 1.5% agar. Puromycin was added, when needed, at a final concentration of 2.5 μg/ml. Lysogeny broth (LB) was used to culture *Escherichia coli* DH10B at 37 °C with shaking at 200 rpm and supplemented with 100 μg/ml ampicillin when needed. For growth on solid medium, LB was solidified with 1.5% agar.

*S. acidocaldarius* MW001 were grown at pH 3.5 and 75 °C in Brock medium supplemented with 0.1% NZ-Amine, 0.2% sucrose, and 10 μg/mL uracil^56^. For growth on solid medium, above medium was solidified with 0.6% gelrite.

### Plasmid construction

Plasmids and primers used in this study are listed in Supplementary Tables 2–3. Primers were synthesized from GENEWIZ company (Suzhou, China). The DNeasy Blood and Tissue Kit (Qiagen) was used to isolate genomic DNA. According to the manufacturer’s protocol, PCR was performed using Taq DNA polymerase or Phusion high-fidelity DNA Polymerase (New England Biolabs). The FastPure Plasmid Mini Kit (Vazyme) was used for the isolation of *E. coli* plasmid DNA. DNA fragments were purified by the FastPure Gel DNA Extraction Mini kit (Vazyme) during cloning procedures. The ClonExpress One Step Cloning Kit (Vazyme) was used for the construction of the plasmids via stepwise Gibson assembly. DNA sequences were confirmed by sequencing at GENEWIZ (Suzhou, China).

To construct the *saci*_*0703* deletion plasmids, 600 bp of the *saci_0703* upstream and downstream regions were PCR amplified from *Sulfolobus acidocaldarius* MW001 genomic DNA with primers Z034F/R and Z035F/R, respectively, and inserted into the *NcoI* and *BamHI* restriction sites of pSVA407^56^ via the stepwise Gibson assembly to yield plasmid pSVA407-*saci_0703*UD.

To construct expression plasmids in *Methanococcus maripaludis* S001, *saci_0703*, *ma_1486* and *ma_1114* were amplified from *S. acidocaldarius* MW001 and *Methanosarcina acetivorans* C2A genomic DNA by PCR respectively, and inserted into the *AfeI* and *NotI* sites of expression plasmid pMEV4^57^ via the stepwise Gibson assembly, to yield expression plasmids pMEV4-*saci_0703*, pMEV4-*ma_1486* and pMEV4-*ma_1114*. The *maeo_0574* DNA fragment was synthesized by GENEWIZ company and cloned into the plasmid pMEV4 by the same procedure.

### Transformation of plasmids into host cells

Electrocompetent *E. coli* DH10B cells were transformed by electroporation using a Micro-Pulser Electroporator (BioRad) with the program Ec1(1.8 kv, 1 pulse for a 0.1-cm cuvette) and the transformants were selected on 100 μg/ml ampicillin. The constructed expression plasmids were transformed into *M. maripaludis* S001 using the polyethylene glycol (PEG)-mediated transformation approach^58^ and selected by plating on puromycin (2.5 μg/ml) McF agar medium, and confirmed via PCR amplification using primers P1F/R. Transformation of deletion plasmid pSVA407-*saci_0703*UD into *S. acidocaldarius* MW001 was performed by electroporation using a Micro-Pulser Electroporator (BioRad) with the program Ec1(1.8 kv, 1 pulse for a 0.1 cm cuvette) and the transformants were selected on Brock medium plate supplemented with 0.1% NZ-Amine and 0.2% sucrose 19.

### Lipid extraction and analyses

Cultures of *M. maripaludis* S001 transformants (100 mL) were incubated to stationary phase at 37 °C, harvested by centrifugation at 10,000 × *g* for 10 min, and pellets were stored at −80 °C before extraction. Frozen cell pellets were freeze-dried, and ~30 mg of dried mass was weighed and ground with a laboratory spatula. An internal standard, C46 GTGT (118 ng)^59^, was added to the ground pellets, before acid hydrolysis in 40 mL capped glass vials with 5 mL 10% hydrochloric acid (HCl) in methanol (MeOH) (vol/vol) at 70 °C overnight. In all, 10 mL of dichloromethane (DCM) and 10 mL of nanopure water were added to the acid-treated mixture, and lipids in the mixture was extracted with DCM for three times in a 50-mL Teflon tube. The samples were centrifuged for 10 min at 2800 × *g* to separate the aqueous and organic phases. The bottom organic phase layer was transferred to a collection glass vial. The organic phases were combined and filtered through a 0.45-μm PTFE filter and dried under N_2_ gas.

Lipid analysis was performed on an Agilent 1200 series high-performance liquid chromatography -atmospheric pressure chemical ionization- 6460A triple quadruple mass spectrometer (HPLC-APCI-MS^2^). Compound separation was achieved with an Alltech Prevail Cyano column (150 mm × 2.1 mm, 3 μm) at a constant flow rate of 0.2 mL/min throughout. The elution gradient followed Schouten et al.^60^ with some modifications. Lipids were eluted isocratically for the first 5 min with 90% A and 10% B, where A = *n*-hexane and B = *n*-hexane/isopropanol (9:1, *v*/*v*), followed by the linear gradient: 90/10 A/B to 82/18 A/B from 5 to 45 min. Finally, 100% B (10 min) was used to wash the column, and then 90/10 A/B to equilibrate the column. Archaeal ether lipids were scanned in a single ion monitoring (SIM) mode, targeting *m*/*z* 653, 651, 1304, 1302, 1300, 1298, 1296, 1294, 1292. APCI settings were as follows: nebulizer pressure 60 psi, vaporizer temperature 400 °C, drying gas flow 6 L/min and temperature 200 °C, capillary voltage 3500 V, corona current 5 μA (~3200 V). Data were analyzed by Agilent MassHunter software (B.07.00 version).

To obtain the MS^2^ spectra and the fragmentation features of targeted compounds, several samples containing macrocyclic archaeol and GDGT-0 were selected and analyzed with an Agilent 1260 series HPLC system coupled to an Agilent 6530B quadrupole time-of-flight (qTOF) mass spectrometer through an APCI interface. The elution gradient followed Yang et al. ^61^. Separation of archaeal ether lipids was performed with two silica columns (150 mm × 2.1 mm, 1.9 μm, Thermo Finnigan; USA) maintained at 40 °C. Archaeal ether lipids were eluted isocratically for the first 5 min with 84% A and 16% B, where A = *n*-hexane and B = ethyl acetate (EtOA). Linear gradients, i.e., 84/16 A/B to 82/18 A/B from 5 to 30 min, and 82/18 A/B to 100% B from 30 to 31 min were used. 100% B was held for 4 min and then changed to 16% B in 1 min. Finally, 84/16 A/B was held for 14 min. The APCI settings were as follows: drying gas temperature 200 °C, vaporizer temperature 400 °C, the flow rate of drying gas 6 L/min, nebulizer pressure 60 psi, capillary voltage 3500 V, and corona current 5 μA. To generate the MS^2^ spectra, the collision energy for archaeol, GDGT-0, macrocyclic archaeol, and GTGT-0 were 0, 35, 15, and 35, respectively. The qTOF settings were capillary voltage 3.5 kV, corona current 5 μA, fragmentor voltage 200 V, skimmer voltage 60 V, and octopole voltage 750 V. In the auto MS/MS scanning mode, the mass of [M + H]^+^ ions scanned in MS^1^ ranged from *m/z* 100 to 1700. In MS^2^ experiments, the scanned mass of product ions ranged from *m/z* 50 to 1700.

Macrocyclic archaeol was purified by a semi-preparative HPLC on an Agilent series 1290 HPLC equipped with a fraction collector. Compound separation was performed with an Alltech Prevail Cyano column (150 mm × 2.1 mm, 3 μm) and the same elution gradient used on HPLC-QQQ-MS. The fraction containing purified macrocyclic archaeol was transferred into a vial and dried with N_2_ gas. Boron tribromide (BBr_3_) (0.5 mL, 1 M in DCM; Sigma-Aldrich, USA) was added and the vial was sealed. The vial was heated at 60 °C for 2 h, and residual solvents were removed in a gentle stream of N_2_ gas. The bromides were then reduced to alkanes by superhydride (0.5 mL, lithium triethylborohydride (LiEt_3_BH), in DCM, Sigma-Aldrich, USA) in a sealed vial with an Argon atmosphere at 60 °C for 2 h. Water was added to quench the reaction. The resulting alkanes were extracted for six times with *n*-hexane. The alkanes were analyzed by a Thermo Finnigan Trace 1300 gas chromatography coupled to an ISQ 7000 mass spectrometer (GC-MS) equipped with a DB-5MS silica capillary column (60 m × 0.25 mm × 0.25 μm) with helium as carrier gas. The initial oven temperature is 70 °C, followed by an increase to 210 °C at 10 °C/min and an increase to 310 °C at 3 °C/min. The final temperature was held at 310 °C for 26 min. The mass spectrometer conditions were as follows: EI energy 70 eV and the auxiliary temperature of GC and MS was 310 °C. Biphytane was identified based on the mass spectra and previous studies^62,63^.

### Bioinformatics analyses

Tetraether synthase (Tes) homologs were identified using BLASTP searches (*e*-value < 1e^−50^, identity >20%) of the archaeal database on JGI IMG (https://img.jgi.doe.gov) with the Tes (MA_1486) sequence of *Methanosarcina acetivorans* C2A as the search query. A total of 2073 archaeal genomes were searched including 889 isolate genomes, 792 metagenome acquired genomes (MAGs), and 392 single-cell acquired genomes (SAGs). We also searched Tes homologs with 13,919 finished and draft bacterial genomes in the JGI IMG database using the same query and parameters above. Total 1104 archaeal and 456 bacterial Tes homologs were retrieved from multiple phyla, respectively.

Sequences greater than 400 amino acids (aa) were selected and clustered at 90% similarity through CD-HIT version 4.7^64^, resulting in unique 426 archaeal and 185 bacterial Tes homologs from the initial genome searches, respectively. The outgroup for archaeal Tes phylogenetic tree consisted of 41 archaeal GDGT ring synthase (Grs) homologs and the outgroup for that of bacteria consisted of 5 archaeal Tes homologs. Protein sequences were aligned via MUSCLE version 3.8.31^65^ and automatically trimmed via trimAl version 1.4^66^ with the outgroup included. Maximum likelihood trees were built by RAxML version 8.2.12^67^ using the CAT model of rate heterogeneity with the LG substitution matrix and 1000 bootstrap iterations. Interactive tree of life (iTOL) was used for tree visualization and annotation (https://itol.embl.de/)^68^.

BLASTP searches of Tes homologs were also carried out in the Non-redundant protein sequences (nr) database on NCBI (https://www.ncbi.nlm.nih.gov/) with the same query described above. Parameters were set as follows: word size = 6, BLOSUM 62, Expect threshold = 10. Low complexity regions were filtered and lower case letters were masked. The specific MAGs datasets of Marine Group II (taxid: 133814), Marine Group III (taxid:347538), Acidobacteriota (taxid:57723), Proteobacteria (taxid:1224), Terrabacteria group (taxid:1783272), FCB group (Fibrobacteres−Chlorobi−Bacteroidetes, taxid:1783270), PVC group (Planctomycetes−Verrucomicrobia−Chlamydiae, taxid:1783257), CPR group (Candidate Phyla Radiation, taxid:1618338−1618340; Patescibacteria group, taxid:1783273) were assigned respectively during the BLASTP searches to identify the Tes homologs (*e*-value < 1e^−50^, identity >20%, length >400 aa) in different archaeal and bacterial communities. The results were summarized in Fig. 4 and Supplementary Table 2.

### Reporting summary

Further information on research design is available in the Nature Research Reporting Summary linked to this article.

## Supplementary information

Reporting Summary

Supplementary information

## Data Availability

The data supporting the findings from this study are available in this article and its Supplementary Information file. Source data are provided with this paper. Gene and protein sequences were obtained from gene database on JGI IMG (https://img.jgi.doe.gov) and on NCBI (https://www.ncbi.nlm.nih.gov/). Source data are provided with this paper.

## Source data

Source Data
