# Supplementary material for: Identification of a protein responsible for the synthesis of archaeal membrane-spanning GDGT lipids
Source: Nat Commun. 2022 Mar 22;13:1545. doi: 10.1038/s41467-022-29264-x (PMC8941075; doi:10.1038/s41467-022-29264-x)
Supplement: Supplementary file 2 — Supplementary information [file 41467_2022_29264_MOESM2_ESM.pdf]

Supplementary Information

Identification of a protein responsible for the synthesis of archaeal membrane-spanning GDGT lipids

Zhirui Zeng*, Huahui Chen, Huan Yang, Yufei Chen, Wei Yang, Xi Feng, Hongye Pei, and Paula V. Welander*

Corresponding Authors: Zhirui Zeng, Paula V. Welander

Emails: zengzr@sustech.edu.cn, welander@stanford.edu

Contains:

Supplementary Fig. 1-5
Supplementary Table 1-3
Supplementary References

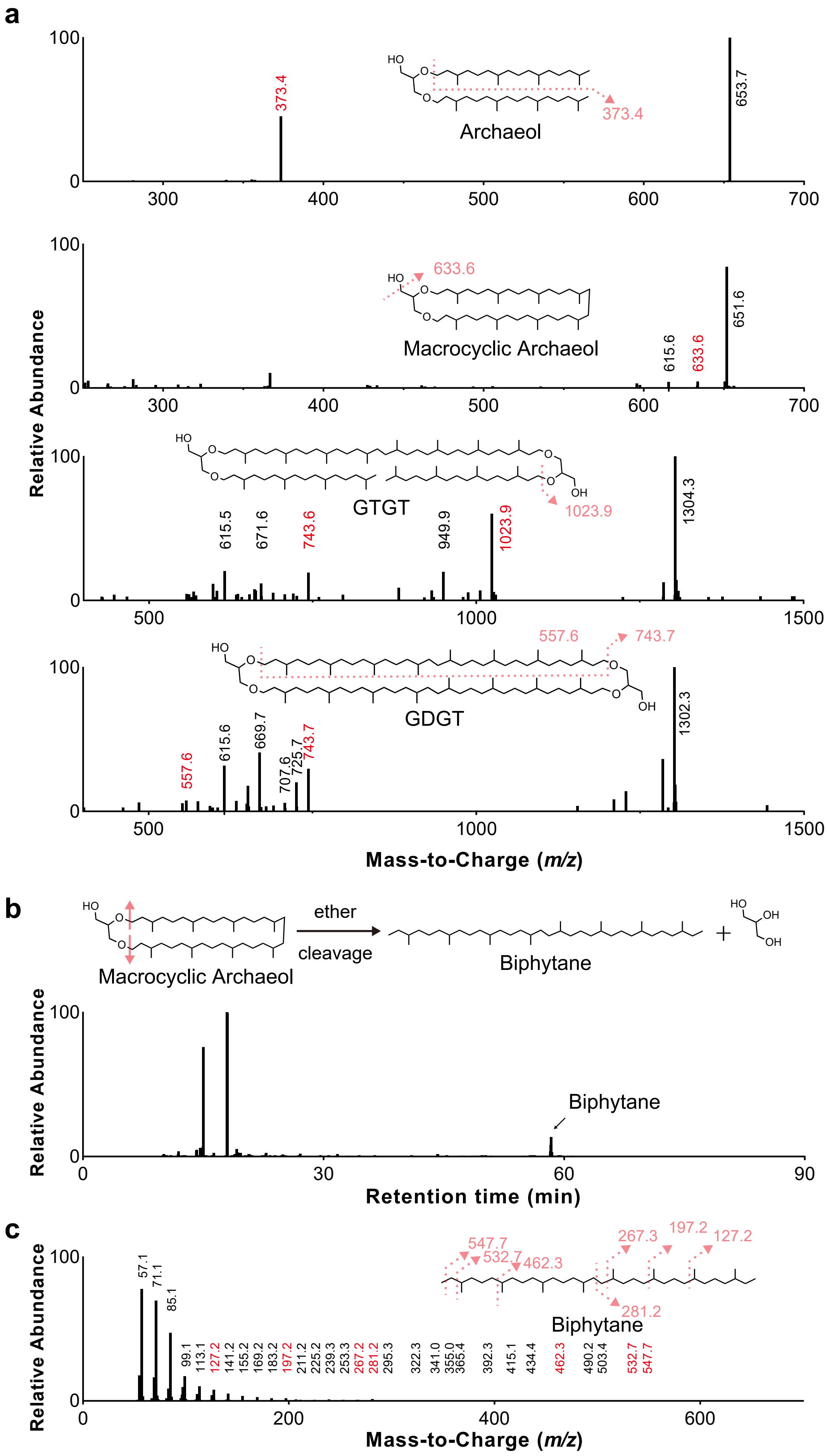

Supplementary Fig. 1 Mass spectra of lipids from heterologous expression of Tes (Maeo_0574) in *M. maripaludis*. **a**, Mass-mass spectra of lipid extracts from expression of pMEV4-*maeo_0574* in *M. maripaludis*. **b**, The LC-MS extracted ion chromatograms of biphytane released from ether cleavage of purified macrocyclic archaeol. **c**, Mass spectra of above selected biphytane, indicating macrocyclic archaeol is one product of Tes expression. Source data are provided as a Source Data file.

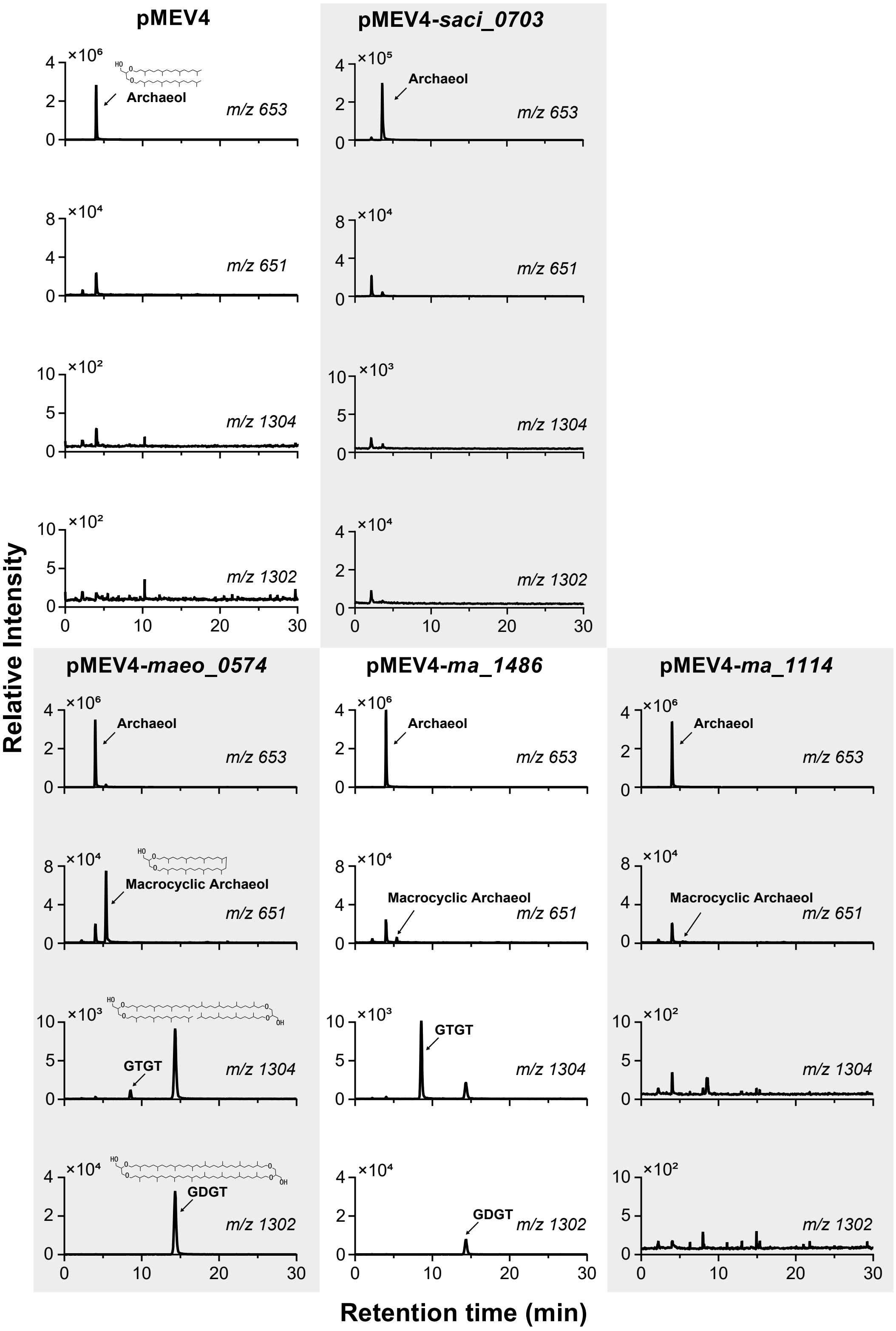

Supplementary Fig. 2 The formation of lipids from heterologous expression of different Tes homologous genes in *M. maripaludis*. LC-MS extracted ion chromatograms of lipid extracts from *M. maripaludis* with empty plasmid pMEV4 or with *saci_0703*, *maeo_0574*, *ma_1486*, or *ma_1114* expressed on plasmid pMEV4. The expression of *maeo_0574* or *ma_1486* resulted in the formation of GDGT, GTGT and macrocyclic archaeol, while the expression of *MA_1114* only produced small amount of macrocyclic archaeol. Source data are provided as a Source Data file.

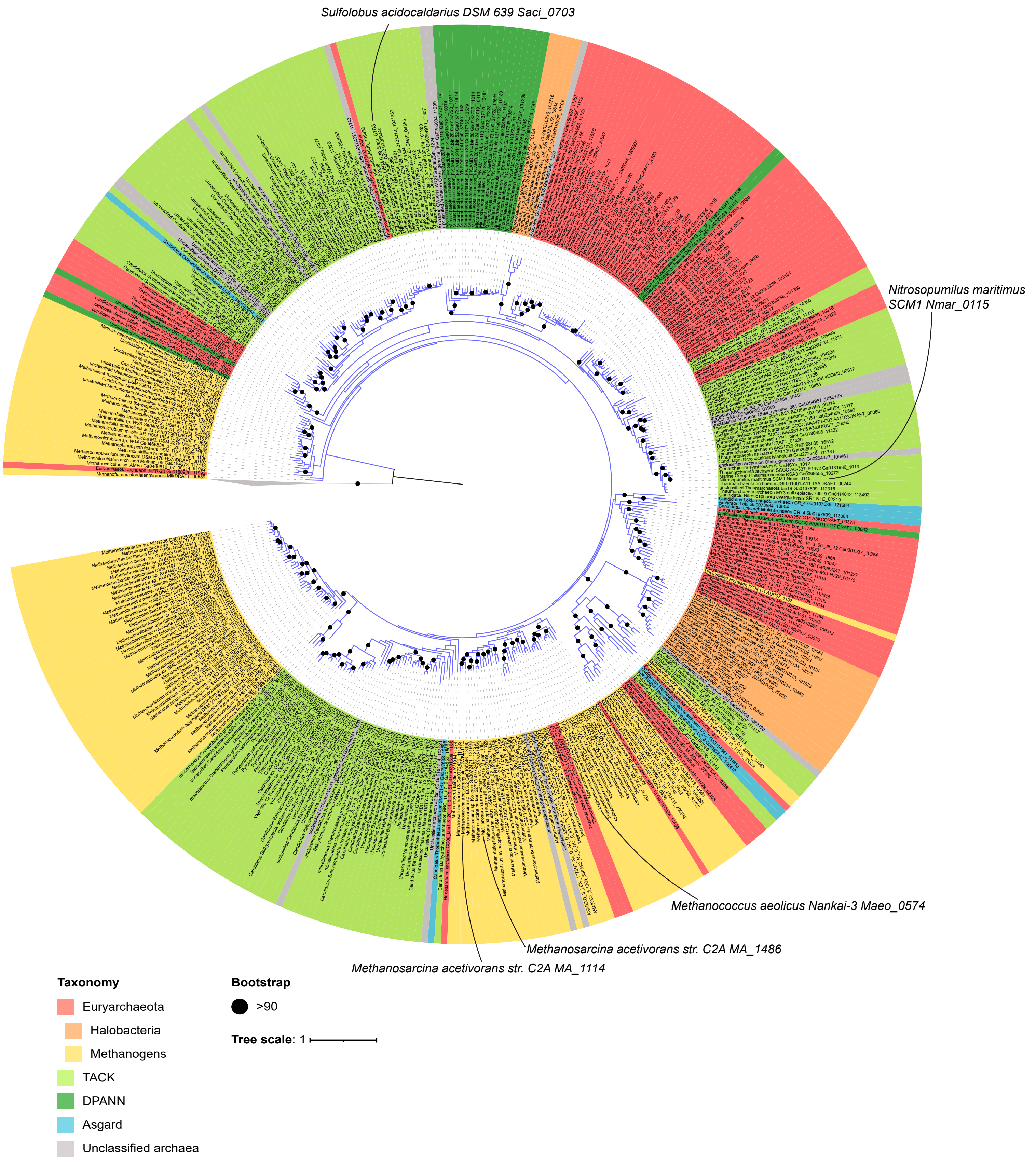

Supplementary Fig. 3 Phylogeny of Tes proteins in Archaea domain. Maximum-likelihood tree was generated using RAxML 8.2.12 with 1,000 bootstrap iterations based on the alignment of 426 unique archaeal Tes homologs from 2,073 archaeal genomes in the JGI IMG database and 41 archaeal Grs homologs as an outgroup. Archaeal superphyla covering Asgard, TACK, Euryarchaeota and DPANN are annotated with different colors in the tree. Halobacteria and methane-related archaea belonging to Euryarchaeota are also highlighted. Black circles indicate branches having bootstrap values ≥ 90. Source data are provided as a Source Data file.

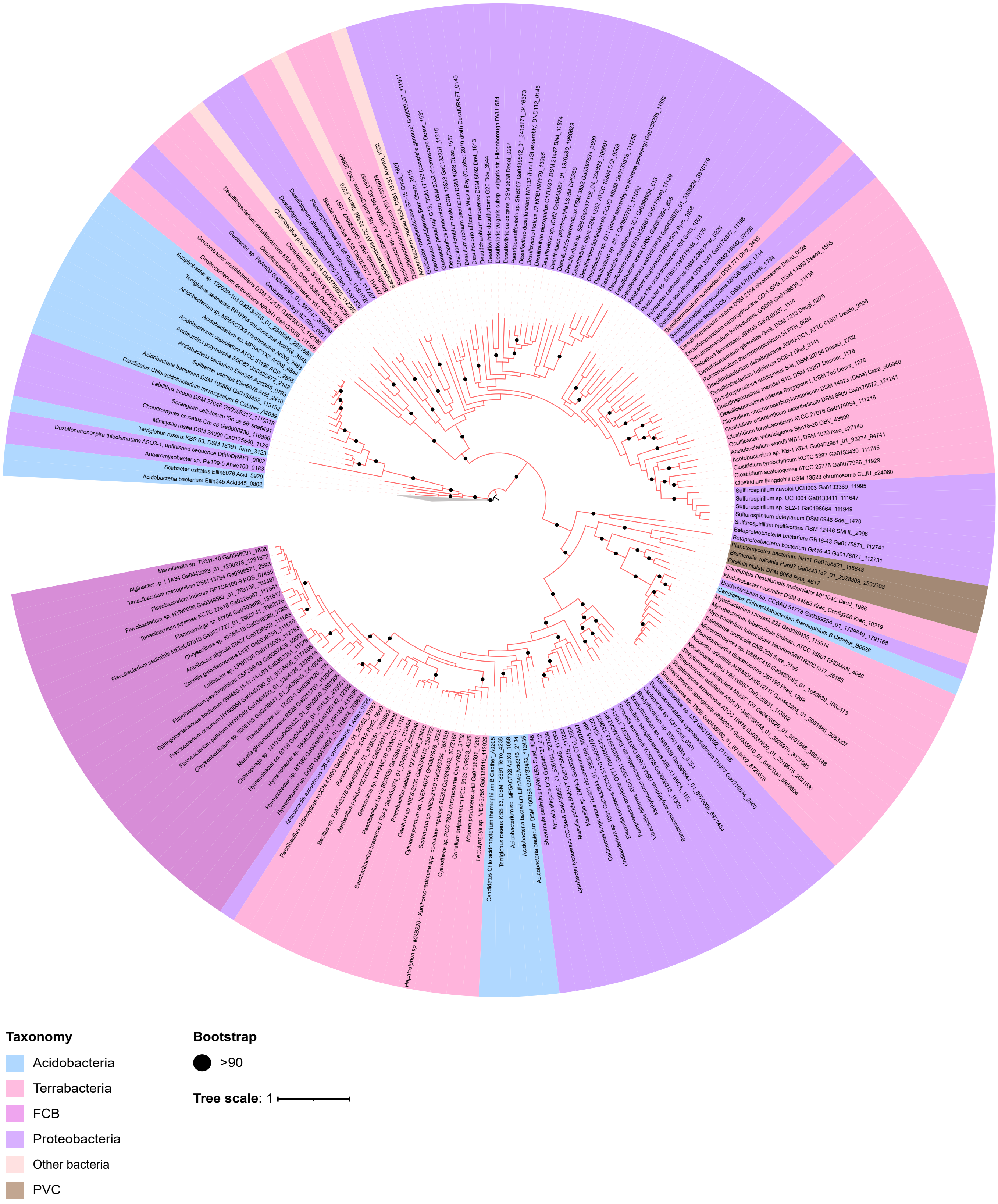

Supplementary Fig. 4 Phylogeny of Tes proteins in Bacteria domain. Maximum-likelihood tree was computed using RAxML 8.2.12 with 1,000 bootstrap iterations based on the alignment of 185 unique bacterial Tes homologs from 13919 bacterial genomes in the JGI IMG database and 5 archaeal Grs homologs as an outgroup. Bacterial phyla of Proteobacteria and Acidobacteria as well as superphyla of Terrabacteria, FCB (Fibrobacteres-Chlorobi-Bacteroidetes) and PVC (Planctomycetes-Verrucomicrobia-Chlamydiae) are highlighted. Bacterial phyla that do not belong to these groups are assigned "Other bacteria" in the tree, including Synergistetes and Fusobacteria. Black circles indicate branches having bootstrap values ≥ 90. Source data are provided as a Source Data file.

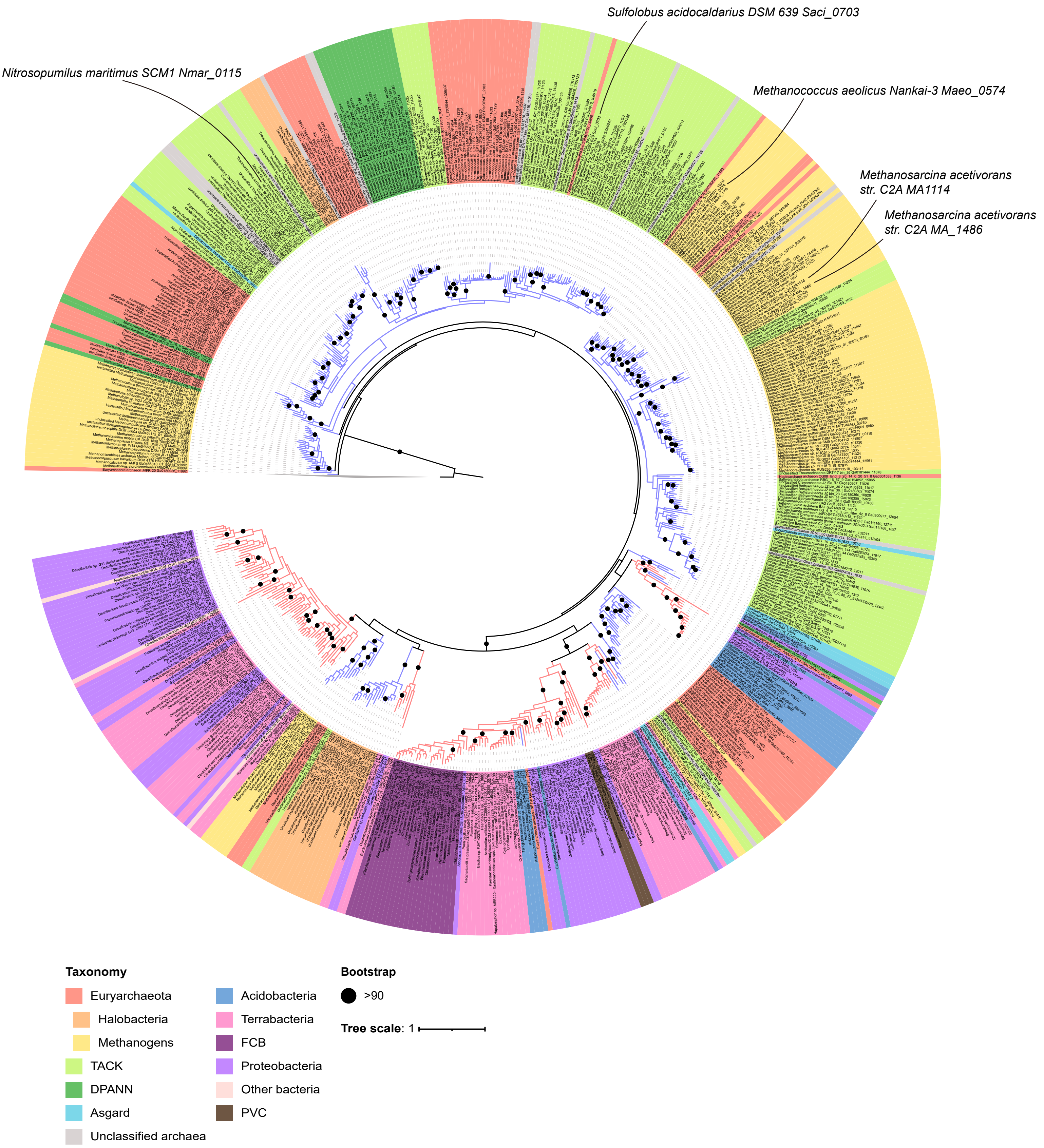

Supplementary Fig. 5 Phylogeny of Tes proteins in both Archaea and Bacteria domains. Maximum-likelihood tree was computed using RAxML 8.2.12 with 1,000 bootstrap iterations based on the alignment of total 652 amino acid sequences covering 426 unique archaeal Tes homologs (Supplementary Fig. 3), 185 unique bacterial Tes homologs (Supplementary Fig. 4) and 41 archaeal Grs homologs as an outgroup. Blue branches indicate phylogenetic lineages of archaea and red branches indicate those of bacteria. The colors of superphyla and phyla are assigned following Supplementary Fig. 3 and Supplementary Fig. 4. Black circles indicate branches having bootstrap values ≥ 90. Source data are provided as a Source Data file.

Supplementary Table 1. Strains used in this study

Strains		Genotype	Source or reference
Escherichia coli	DH10B	F^{-} *endA1 recA1 galE15 galK16 nup GrpsL ΔlacX74 Φ80lacZΔM15 araD139 Δ(ara,leu)7697 mcrA Δ(mrr-hsdRMS-mcrBC)*λ^{-}	Paula V. Welander (Stanford University)
	ER1281	F^{-}*glnV44 e14*$^{-}$*(McrA*$^{-}$*) rfbD1 relA1 endA1 spoT1 thi-1 Δ(mcrC- mrr)114::IS10*	New England Biolabs
Methanococcus maripaludis	S001	Expression host containing ORF1 from pURB500 integrated into the *M. maripaludis* S2 genome	(1)
	HH001	S001+ pMEV4-mCherry-pac	This work
	HH002	S001+pMEV4-*ma_1114*	This work
	HH003	S001+pMEV4-*ma_1486*	This work
	HH004	S001+pMEV4-*maeo_0574*	This work
	HH005	S001+pMEV4-*saci_0703*	This work
Sulfolobus acidocaldarius	MW001	*ΔpyrE* (uracil auxotrophic parent strain)	(2)

Supplementary Table 2. Plasmids used in this study

Plasmids	Description	Source or reference
pMEV4-mCherry-pac	*M. maripaludis* S001 expression plasmid containing the puromycin and ampicillin resistance.	(3)
pMEV4-*saci_0703*	*M. maripaludis* S001 expression plasmid containing *saci_0703* gene, which was amplified by PCR with primers P5F/R and cloned into the *AfeI* and *NotI* site of pMEV4 plasmid.	This work
pMEV4-*ma_1114*	*M. maripaludis* S001 expression plasmid containing *ma_1114* gene, which was amplified by PCR with primers P2F/R and cloned into the *AfeI* and *NotI* site of pMEV4 plasmid.	This work
pMEV4-*ma_1486*	*M. maripaludis* S001 expression plasmid containing *ma_1486* gene, which was amplified by PCR with primers P3F/R and cloned into the *AfeI* and *NotI* site of pMEV4 plasmid.	This work
pMEV4-*maeo_0574*	*M. maripaludis* S001 expression plasmid containing *maeo_0574* gene, which was amplified by PCR with primers P4F/R and cloned into the *AfeI* and *NotI* site of pMEV4 plasmid.	This work
pSVA407	*S. acidocaldarius* deletion plasmid.	(2)
pSVA407-*saci_0703*UD	*S. acidocaldarius* deletion plasmid containing *saci_0703* upstream and downstream regions for *saci_0703* deletion. The upstream and downstream regions were amplified by PCR with primers Z034F/R, and Z035F/R, respectively, and cloned into the *NcoI* and *BamHI* sites of pSVA407 plasmid.	This work

Supplementary Table 3. Primers used in this study

**Primers**	**Sequence (5' to 3')**	**Notes**
P1F	GGCTTATGAAATTTGTTAAAATTTAGC	pMEV4 plasmid cloning check
P1R	TACCCAAATATTTCAATGAATATTTAG	
P2F	TAACTAATACTAGAGTGCAGGTAGCGCTATGGGATCAGCAGGTGTTTAC	*ma_1114* cloning into plasmid pMEV4
P2R	GAATAGGGCGTTTTTATCTGCAGCCTATGCTTCAGAATAATAAGGCACTG	
P3F	TAACTAATACTAGAGTGCAGGTAGCGCTATGTATGATTTTTTGGAGAGTGTATTAATG	*ma_1486* cloning into plasmid pMEV4
P3R	GAATAGGGCGTTTTTATCTGCAGCTTAATGGACTTCAAAAGATTCCTCCTC	
P4F	TAACTAATACTAGAGTGCAGGTAGCGCTATGAAAACCATATCTTTATGCCCTAC	*maeo_0574* cloning into plasmid pMEV4
P4R	GAATAGGGCGTTTTTATCTGCAGCTTATTTTTTTCTTTTTTTCCATTCCTCAATAGG	
P5F	ACTAGAGTGCAGGTAGCGCTATGACTGAAACTGTACAAGAG	*saci_0703* cloning into plasmid pMEV4
P5R	GTTTTTATCTGCAGCGGCCGCTTAGCTAGATGTAAAATTC	
Z034F	CCCGACGTCGCATGCTCCCGGCCGCGTAGATAATATAAAAAGAGGGGACAAAGTA	*saci_0703* upstream cloning forward
Z034R	TTATAAAGCTCTCCTTGCTCACATATTCTGTCTTATCACT	*saci_0703* upstream cloning reverse
Z035F	AGTGATAAGACAGAATATGTGAGCAAGGAGAGCTTTATAA	*saci_0703* downstream cloning forward
Z035R	TACTAGAACTGCTCAAACCTAGGTCAGAATAGATAATAAAAACCTTCTGATTATAGA	*saci_0703* downstream cloning reverse

Supplementary References

1. Walters, A. D., Smith, S. E. & Chong, J. Shuttle vector system for *Methanococcus maripaludis* with improved transformation efficiency. *Appl. Environ. Microbiol.* **77**, 2549–51 (2011).

2. Michaela, W. *et al.* Versatile genetic tool box for the Crenarchaeote *Sulfolobus acidocaldarius*. *Front. Microbiol.* **3**, 1-12 (2012).

3. Zhe, L., Jain, R., Smith, P., Fetchko, T. & Whitman, W. B. Engineering the autotroph *Methanococcus maripaludis* for geraniol production. *ACS Synth. Biol.* **5**, 577-581 (2016).
